# Brain-specific NRSF deficiency aggravates dopaminergic neurodegeneration and impairs neurogenesis in the MPTP mouse model of Parkinson’s disease

**DOI:** 10.18632/aging.101979

**Published:** 2019-05-30

**Authors:** Dongping Huang, Qing Li, Yi Wang, Zhaolin Liu, Zishan Wang, Heng Li, Jinghui Wang, Jing Su, Yuanyuan Ma, Mei Yu, Jian Fei, Fang Huang

**Affiliations:** 1 Department of Translational Neuroscience, Jing’ an District Centre Hospital of Shanghai, State Key Laboratory of Medical Neurobiology and MOE Frontiers Center for Brain Science, Institutes of Brain Science, Fudan University, Shanghai 200032, China; 2 Department of Neurology, Huashan Hospital, Fudan University, Shanghai 200040, China; 3 School of Life Science and Technology, Tongji University, Shanghai 200092, China; 4 Shanghai Engineering Research Center for Model Organisms, Shanghai Model Organisms Center, INC., Shanghai 201203, China; * Equal contribution

**Keywords:** Parkinson’s disease, neurodegeneration, NRSF/REST, astrocyte activation, neuroinflammation

## Abstract

Degeneration of the dopaminergic neurons in the substantia nigra and the resultant dopamine depletion from the striatum are the hallmarks of Parkinson’s disease (PD) and are responsible for the disease’s cardinal motor symptoms. The transcriptional repressor Neuron-Restrictive Silencer Factor (NRSF), also known as RE1-Silencing Transcription Factor (REST), was originally identified as a negative regulator of neuron-specific genes in non-neuronal cells. Our previous study showed that mice deficient in neuronal NRSF/REST expression were more vulnerable to the noxious effects of the dopaminergic neurotoxin MPTP. Here, we found that brain-specific deletion of *NRSF/REST* led to more severe damages to the nigrostriatal pathway and long-lasting behavioral impairments in mice challenged with MPTP. Moreover, compared to wild-type controls, these mice showed increased neurogenesis shortly after MPTP exposure, but reduced neurogenesis later on. These results suggest that NRSF/REST acts as a negative modulator of neurogenesis and a pro-survival factor of neural stem cells under both normal conditions and during the course of PD.

## INTRODUCTION

Progressive loss of dopaminergic neurons in the substantia nigra par compacta (SNpc) with resultant dopamine depletion in the striatum characterizes Parkinson’s disease (PD) and accounts for the disease’s cardinal motor symptoms [1]. Current therapeutic strategies are mostly based on pharmacological enhancement of dopaminergic neurotransmission. Such approaches have several long-term side effects, including dyskinesia and response fluctuations, which limits their use [2]. The discovery of neurogenesis in the adult mammalian brain has opened up new possibilities for the therapeutics of PD [2, 3].

The evidence for adult neurogenesis stems from the discovery of neuronal precursor cells (NPCs) in the subventricular zone (SVZ) lining the lateral ventricles and the subgranular zone (SGZ) of the dentate gyrus in the hippocampus, that retain the capacity of producing new neurons both in health and disease. This phenomenon was first described in 1962 by J. Altman in rats [4, 5]. Evidence that new neurons are formed in the adult human brain came in 1998, when P.S. Eriksson and colleagues injected bromodeoxyuridine (BrdU) to cancer patients to track dividing cells, and postmortem histology revealed newly generated, BrdU-positive neurons in the hippocampus [6]. Today, research efforts to understand and control neurogenesis are opening exciting new possibilities for the treatment of neurodegenerative disease. Indeed, reparative neurogenesis has been demonstrated in animal models of ischemic stroke, amyotrophic lateral sclerosis, and epilepsy, among others [7–10]. Over the past few years, scientists assessed a massive set of genes, miRNAs, and transcription factors to evaluate their potential influence on neurogenesis and aid the development of new therapies for neurodegenerative diseases.

Experimental evidence has shown that the Neuron-Restrictive Silencer Factor (NRSF), also known as RE1-Silencing Transcription Factor (REST), plays an important role in neurogenesis. Conditional knockout of *NRSF* results in precocious activation of quiescent neural progenitors and reduced neurogenesis over time [11, 12]. In addition, Kim *et al.* found NRSF regulates non-cell-autonomous neuronal differentiation and maturation of neural progenitor cells via secretogranin II (Scg-2) [13]. On the other hand, Covey *et al* found NPCs lacking NRSF display significantly reduced self-renewal capacity owing to reduced cell cycle kinetics and precocious neuronal differentiation [14].

NRSF was initially considered to be a negative regulator of neuron-specific genes in non-neuronal cells [15]. NRSF is highly expressed throughout early development, where it represses the expression of neuronal genes and is involved in the transcriptional silencing of neuronal gene promoters in conjunction with CoRest, which recruits additional silencing machinery, including methyl DNA-binding protein MeCP2, histone deacetylase (HDAC) and histone H3K9 methyltransferase G9a [16]. The expression of NRSF/REST is gradually reduced as embryonic stem cells (ESCs) differentiate into neural stem cells (NSCs), and nearly disappears from mature adult neuronal cells [16]. A disruption of the interaction of NRSF/REST with its target genes has been reported to cause aberrant changes in neuronal gene expression in conditions such as epilepsy, Huntington’s disease, and Down’s syndrome [16–18].

Our previous study also showed that mice deficient in neuronal NRSF expression are more vulnerable to the dopaminergic neurotoxin MPTP, which is used in animal research to mimic the symptomatology of human Parkinson’s disease (PD) [19, 20]. In the present study, the effects of acute MPTP exposure were further assessed in brain-specific *NRSF* conditional knockout mice and littermate wide-type controls [21, 22]. Specifically, we assessed early (7 days) and late (42 days) changes in motor function and neurogenesis and also determined the impact of brain-specific NRSF deficiency on cellular and molecular alterations induced by MPTP.

## RESULTS

### Brain-specific NRSF deletion potentiates PD-associated behavioral deficits in mice challenged with MPTP

We previously showed that injuries in the nigrostriatal pathway induced by the neurotoxin MPTP were more severe in neuronal *NRSF* deficient mice than in WT mice [19, 20]. In this study, we extend those findings through a comprehensive evaluation of neuronal and behavioral alterations resulting from acute MPTP treatment in cKO mice. Mice with *lox* P-flanked alleles of NRSF (*NRSF^fl/fl^*) were bred to *Nestin-Cre* transgenic mice to induce deletion of *NRSF* in the brain [23, 24]. The expected *NRSF* fragment lacking exon 2 was detected by PCR in the cortex, hippocampus, and striatum of *Nestin-Cre*:*NRSF^fl/f^* mice (cKO mice), but not in littermate *NRSF^fl/fl^* mice (WT) (Supplementary Figure 1A). Brain-specific ablation of *NRSF* in cKO mice was confirmed at the transcriptional and translational level. A reduction in *NRSF* transcripts was detected in the brain but not in peripheral tissues of cKO mice by quantitative real-time PCR (Supplementary Figure 1B), while double immunofluorescence against NRSF and NeuN further confirmed *NRSF* depletion in the brains of cKO mice (Supplementary Figure 1C). Of note, a striking reduction of body weight was observed in cKO mice compared with WT littermates (Supplementary Figure 2).

Nissl staining revealed no obvious morphological abnormalities in the brains of cKO mice compared to WT littermates (Supplementary Figure 3). Striatal levels of amino acid neurotransmitters were next characterized by HPLC. Results showed increased levels of GABA in cKO mice (Supplementary Figure 4A), whereas glycine, aspartate, asparagine, glutamate, and glutamine levels did not differ from WT mice (Supplementary Figure 4B–4F). No differences regarding locomotor ability and motor coordination were detected between genotypes in rotarod and open field tests (Supplementary Figure 4G–4I).

**Figure 1:**
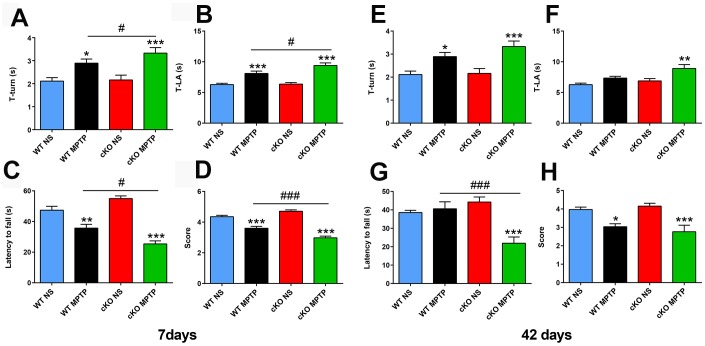
**Deletion of *NRSF* in the brain exacerbates motor defects induced by MPTP.** (**A**–**D**) Behavioral assays at 7 days after saline or MPTP administration. (**A**, **B**) Pole test results. (**A**) Time to turn. (**B**) Total time. N = 18-21. (**C**, **D**) Wire hanging test results. (**C**) Latency time. (**D**) Score. N = 16-19. (**E**–**H**) Behavioral assays at 42 days after saline or MPTP administration. (**E**, **F**) Pole test results. (**E**) Time to turn. (**F**) Total time. N = 9-12. (**G**, **H**) Wire hanging test results. (**G**) Latency time. (**H**) Score. N = 9-11. All data are means ± SEM. Differences were analyzed by one-way ANOVA. *p < 0.05, **p < 0.01, ***p < 0.001, vs saline-treated control; #*p* < 0.05, ### *p* < 0.001, vs MPTP-treated WT mice.

To assess the impact of brain-specific NRSF silencing on the behavioral, cellular, and molecular alterations induced by MPTP, 12-16 weeks old cKO and WT control mice received 4 intraperitoneal injections of either 20 mg/kg MPTP-HCl or saline solution, spaced by 2 h intervals. Early and late PD-associated behavioral deficits were assessed by applying the pole and the wire hanging tests 7 and 42 days after MPTP administration. Pole test results showed obvious motor dysfunction in both WT and cKO mice exposed to MPTP, and this deficit was significantly enhanced in cKO mice (Figure 1A, 1B). In the wire hanging test, MPTP-treated WT and cKO mice both showed shorter fall latency time than saline-treated controls; here again, cKO mice challenged with MPTP scored also significantly lower than MPTP-challenged WT mice (Figure 1C, 1D). To assess whether the behavioral impairment was enduring, mice were tested 42 days after MPTP injection. Results showed that motor deficits were still present in all MPTP-intoxicated animals (Figure 1E), and were again more severe in cKO mice (Figure 1F). Consistently, MPTP-treated cKO mice also scored significantly worse than both MPTP-treated WT and saline-treated mice (Figure 1G, 1H).

### MPTP-induced neurodegeneration is aggravated by NRSF deletion in brain cells

**Figure 2:**
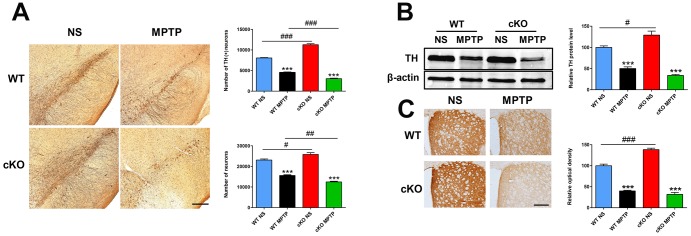
**NRSF deficiency aggravates early damage in the nigrostriatal pathways induced by MPTP.** (**A**) Immunohistochemical staining of TH in the substantia nigra at 7 days after saline or MPTP injection. Scale bar: 200 μm. Statistics for TH- and Nissl-positive neurons are shown in the right panel. N = 5-6. (**B**) Western blot analysis of TH expression in the striatum. Quantification of relative TH expression is shown in the right panel. N = 4. (**C**) Immunohistochemical staining of TH in the striatum. Scale bar: 200 μm. Densitometric analysis of TH staining is shown in the lower right panel. N = 4. All data are means ± SEM. Differences were analyzed by one-way ANOVA. ***p < 0.001, vs saline-treated control; #*p* < 0.05, ##*p*<0.01, ### *p* < 0.001, vs MPTP-treated WT mice.

In previous studies we found that the number of TH- positive (+) neurons in the SN and the density of TH (+) nerve fibers in the striatum were dramatically higher in neuron-specific *NRSF* knockout mice compared to WT mice [19, 20]. These phenotypes reappeared in the brain of the cKO mice used in the present study, as determined by both immunohistochemistry and stereological cell counting (Figure 2). Damage to the nigrostriatal axis, including reductions in the number of TH (+) neurons in the SNpc, striatal TH protein levels, and density of TH (+) nerve fibers, were detected in both WT and cKO mice 7 days after MPTP administration (Figure 2). However, reductions in both total and dopaminergic neuron numbers (Figure 2A) and striatal TH protein levels (*p*=0.012, *vs* MPTP-treated WT mice, by unpaired t test) (Figure 2B) were more severe in MPTP-treated cKO mice. Meanwhile, HPLC analyses of dopamine (DA) and its metabolites DOPAC and HVA showed higher striatal concentrations of DA and DOPAC in cKO compared to WT mice (Table 1). After MPTP injection, striatal levels of DA, DOPAC and HVA were reduced to 24.8%, 63.3% and 75.8% of respective baseline levels in WT mice, while more marked decreases to 9.4%, 29.8% and 47.2% respectively were measured in cKO mice. In addition, the DOPAC to DA ratio was higher in both WT and cKO mice challenged with MPTP than in saline-treated control mice. Meanwhile, in MPTP-treated cKO mice HVA levels and the HVA to DA ratio were significantly decreased and increased, respectively, compared to saline-treated cKO mice. In turn, the ratio of HVA to DA was significantly increased in MPTP-treated cKO mice compared to MPTP-treated WT mice (Table 1).

**Table 1:** HPLC assays of striatal dopamine, DOPAC, and HVA in MPTP-treated WT and cKO mice.

	**WT Saline (n=6)**	**WT MPTP (n=6)**	**cKO Saline (n=5)**	**cKO MPTP (n=5)**
DA	100±4.23	24.80±5.91^***^	122.73±7.72#	9.39±2.67***
DOPAC	100±6.75	63.29±13.5	209.35±39.2##	29.80±9.74***
DOPAC/DA	100±4.52	264.34±32.4^**^	173.50±29.9	315.62±20.8**
HVA	100±6.91	75.79±8.91	130.59±13.2	47.25±8.1***
HVA/DA	100±5.03	362.63±41.9	109.99±9.6	654.71±118***#

Data are means ± SEM. Differences were analyzed by one-way ANOVA. **p<0.01, ***p<0.001, *vs* saline-treated control; #*p*<0.05, ##*p*<0.01, *vs* MPTP-treated WT mice.

**Figure 3:**
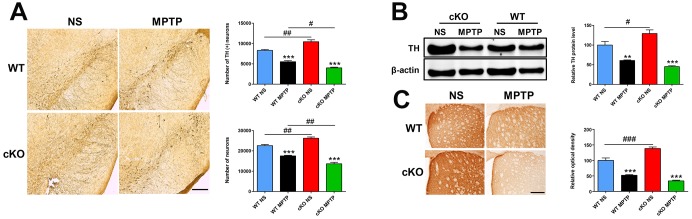
**NRSF deficiency potentiates late damage in the nigrostriatal pathways induced by MPTP.** (**A**) Immunohistochemical staining of TH in the substantia nigra at 42 days after saline or MPTP injection. Scale bar: 200 μm. Statistics for TH- and Nissl-positive neurons are shown in the right panel. N = 4. (**B**) Western blot analysis of TH levels in the striatum. Quantification of relative TH expression is shown in the right panel. N = 4. (**C**) Immunohistochemical staining of TH in the striatum. Scale bar: 200 μm. Densitometric analysis of TH staining is shown in the lower right panel. N = 4. Data are means ± SEM. Data were analyzed by one-way ANOVA. **p < 0.01, ***p < 0.001, vs saline-treated control; #*p* < 0.05, ##*p* < 0.01, ###*p* < 0.001, vs MPTP-treated WT mice.

Late MPTP effects were analyzed at 42 days post-injection. At this time point, sustained damage to the dopaminergic system was detected in both cKO and WT mice (Figure 3). However, compared with the results at 7 days post-injection, WT mice exhibited significant increases in dopaminergic cell numbers (4,541 ± 91 vs 5,536 ± 248, *p* = 0.002), striatal TH protein levels (50.04% ± 4.03% vs 60.82% ± 14.9%, *p* = 0.046) and TH (+) nerve terminals (39.97% ± 1.71% vs 52.04% ± 2.04%, *p* = 0.004). cKO mice also showed partial but significant upregulation in dopaminergic cell numbers (3,028 ± 98 vs 4,014 ± 182, *p* = 0.001) and striatal TH protein levels (33.83% ± 2.1% vs 45.28% ± 2.3%, *p* = 0.01). Thus, although there was a recovery of dopaminergic neurons in cKO mice, loss of both total and dopaminergic neurons, reductions in striatal TH protein levels and TH (+) fiber density (*p*=0.0013 and *p*=0.0011, respectively, *vs* MPTP-treated WT mice, by unpaired t test) remained higher (Figure 3A–3C). These results suggest that despite having higher DA levels in the striatum and more dopaminergic neurons in the SN under baseline conditions, cKO mice are more sensitive to the toxicity of MPTP.

### NRSF deficiency exacerbates astrocyte activation in mice challenged with MPTP

**Figure 4:**
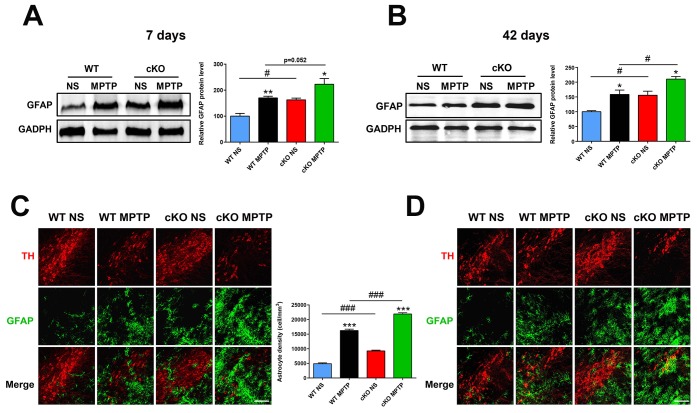
**NRSF deficiency correlates with increased astrocyte activation in the nigrostriatal pathways after MPTP administration.** (**A**) Western blot analysis of striatal GFAP at 7 days after saline or MPTP administration. Quantification of GFAP expression is shown in the right panel. N = 5. (**B**) Immunofluorescence staining of GFAP (green) and TH (red) in the substantia nigra at 7 days after saline or MPTP administration. Scale bar: 100 μm. Statistics for GFAP-positive cells are shown in the right panel. N = 4. (**C**) Western blot analysis of striatal GFAP at 42 days after saline or MPTP administration. Quantification of relative GFAP expression is shown in the right panel. N = 4. (**D**) Immunofluorescence staining of GFAP (green) and TH (red) in the substantia nigra at 42 days after saline or MPTP administration. Scale bar: 100 μm. Data are means ± SEM. Data were analyzed by one-way ANOVA. *P < 0.05, **p < 0.01, ***p < 0.001, vs saline-treated control; #*p* < 0.05, ###*p* < 0.001, vs MPTP-treated WT mice.

Microglia and astrocytes are key modulators of the inflammatory responses that contribute to the progression of PD [25]. Using immunostaining and western blot, we investigated whether NRSF affects glial activation in the MPTP model of PD. One day after MPTP injection, microglial cells in the nigrostriatal pathway were activated in both WT and cKO mice, with no significant differences observed between the two genotypes (Supplementary Figure 5). Meanwhile, higher GFAP expression and more GFAP (+) astrocytes were detected in the nigrostriatal system of untreated cKO mice relative to WT (Figure 4). Seven and 42 days after MPTP injection, astrocytes were obviously activated in the striatum and SN of both WT and cKO mice. Notably, astrocyte activation was more intense in cKO mice, indicated by dramatically elevated levels of striatal GFAP (Figure 4A, 4C), and increased numbers of GFAP (+) astrocytes in both the striatum (Supplementary Figure 6) and the SN (Figure 4B, 4D).

**Figure 5:**
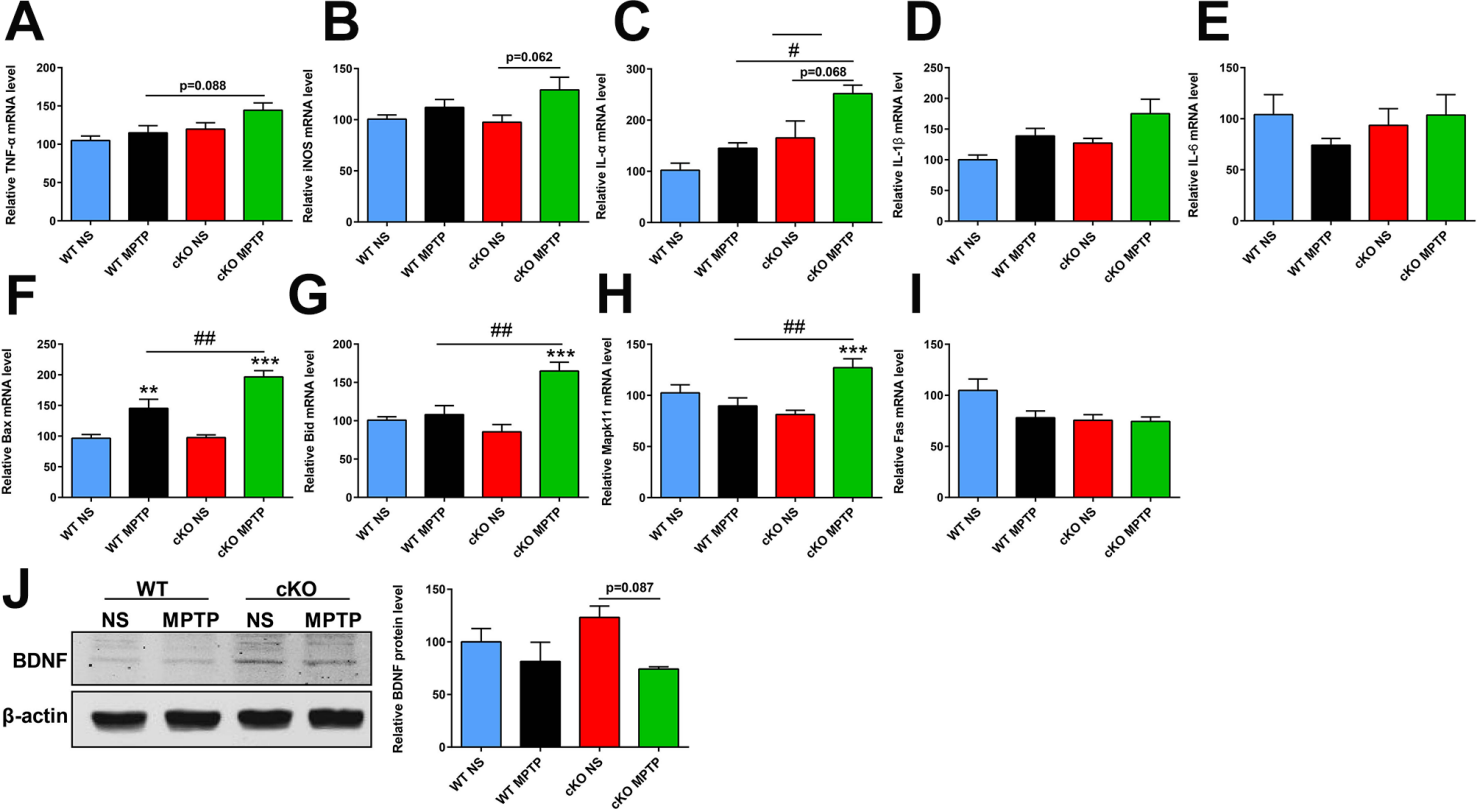
**Transcript levels of selected inflammation- and apoptosis-related genes and BDNF protein expression in the striatum at 7 days after MPTP administration.** (**A**–**E**) Inflammation-related gene transcripts. (**A**) *TNFα*. (**B**) *iNOS*; (**C**) *IL-1α*. (**D**) *IL-1β*. (**E**) *IL-6*. N = 3–11. (**F**–**I**) Apoptosis-related gene transcripts. (**F**) *Bax*. (**G**) *Bid*. (**H**) *Mapk11*. (**I**) *Fas*. N = 7–9. (**J**) Western blot analysis of BNDF expression in the striatum. Quantification of relative BDNF expression levels is shown in the right panel. N = 3. Data are means ± SEM. Data were analyzed by one-way ANOVA. **p < 0.01, ***p < 0.001, vs saline-treated control; #*p* < 0.05, ##*p* < 0.01, *vs* MPTP-treated WT mice.

Chronically activated astrocytes are believed to contribute to PD through production of various pro-inflammatory molecules including tumor necrosis factor alpha (TNF-*α*), inducible nitric oxide synthase (iNOS), interleukin-1α (IL-1α), IL-1*β* and IL-6 [26]. Using quantitative real-time PCR, we found that the expression of *TNF-α* and *IL-1α* in the striatum was increased in MPTP-treated cKO mice compared to MPTP-treated WT mice (*p*=0.088 and *p*=0.035, respectively) (Figure 5A, 5C). Striatal transcripts of *iNOS* and *IL-1α* were also elevated in MPTP-treated cKO mice compared to their saline-treated counterparts (*p*=0.062 and *p*=0.068, respectively) (Figure 5B, 5C). Meanwhile, no changes were observed in *IL-1β* and *IL-6* expression in mice treated with MPTP (Figure 5D, 5E). These results suggest that genetic ablation of *NRSF* triggered more severe neuroinflammation in the MPTP-injured nigrostriatal pathway.

The apoptosis-related genes *Bax*, *Bid*, *Mapk11* and *Fas* are NRSF/REST targets [27], and their transcript levels were comparable between untreated cKO mice and WT littermates. However, upregulated striatal expression of *Bax*, *Bid* and *Mapk11*, but not *Fas*, was observed after MPTP treatment in both WT and cKO mice, and this effect was higher in the latter genotype (Figure 5F–5I). On the other hand, lower BDNF protein levels were found in the striatum of MPTP-treated cKO mice compared to saline-treated cKO counterparts (*p*=0.087) (Figure 5J).

### Brain-specific NRSF deletion transiently accelerates neurogenesis after exposure to MPTP

**Figure 6:**
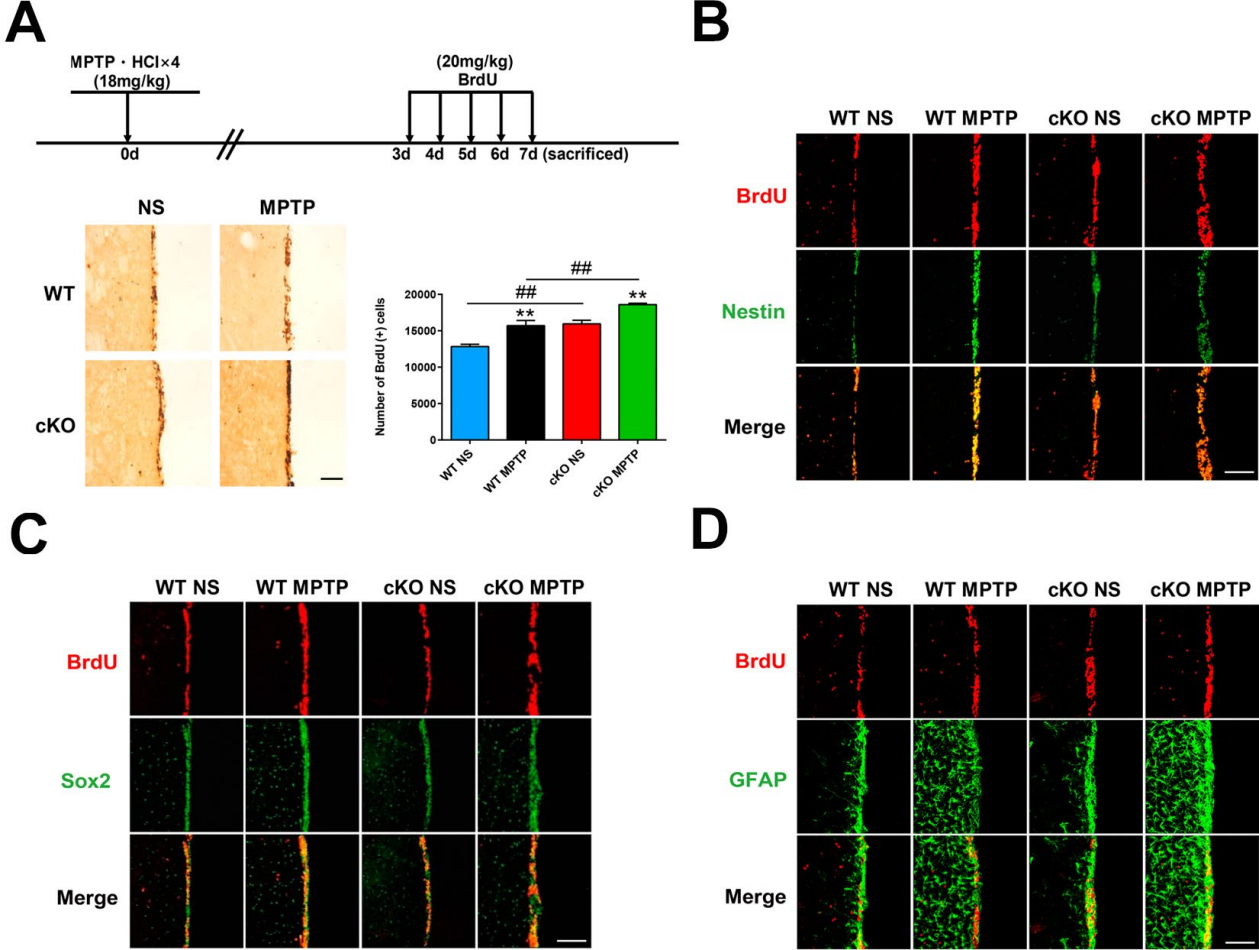
**Early assessment of neurogenesis.** (**A**) Immunohistochemical staining of BrdU in the SVZ, 7 days after MPTP/saline treatment. Scale bar: 200 μm. Quantification of BrdU-positive cells is shown in the right panel. Data are means ± SEM. Data were analyzed by one-way ANOVA. **p < 0.01, *vs* saline-treated control; ##*p* < 0.01, *vs* MPTP-treated WT mice. N = 3–4. (**B**–**D**) Immunofluorescence staining of BrdU (red) and Nestin, Sox2, or GFAP (green) in the SVZ. Scale bar: 100 μm.

The presence of adult neurogenesis in PD is much debated. To investigate the impact of NRSF deficiency on neurogenesis in the MPTP mouse model of PD, BrdU was injected over a 5-day period beginning on day 3 following MPTP treatment, and then sacrificed. Microscopic examination showed that the number of BrdU (+) cells in the SVZ was significantly higher in saline-treated cKO mice than in WT mice (Figure 6A). MPTP treatment further increased the number of BrdU (+) cells in the SVZ of both WT and cKO mice, but the increase was much larger in the latter (Figure 6A). To investigate the identity of BrdU (+) cells, parallel detection of the neural stem cell markers nestin and Sox2, and GFAP, was carried out by immunofluorescence (Figure 6B–6D). To confirm the above results, a second regimen of BrdU incorporation was adopted. BrdU was injected four times daily at 2 h intervals 7 days after the last MPTP injection, and mice were sacrificed for BrdU expression analysis 2 h later. MPTP insult increased the incorporation of BrdU into cells in the SVZ (Supplementary Figure 7A). BrdU (+) cells in the SVZ compartment showed nestin, Sox2, or GFAP immunoreactivity (Supplementary Figure 7B–7D).

### NRSF knockout decreases long-term survival of proliferating neural stem/progenitor cells and severely impairs adult neurogenesis

**Figure 7:**
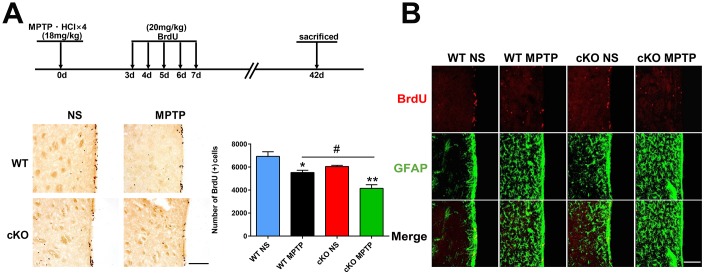
**Late assessment of neurogenesis.** (**A**) Immunohistochemical staining of BrdU in the SVZ, 42 days after MPTP/saline treatment. Scale bar: 200 μm. Quantification of BrdU-positive cells is shown in the right panel. Data are means ± SEM. Data were analyzed by one-way ANOVA. *p < 0.05, **p < 0.01, vs saline-treated control; #*p* < 0.05, vs MPTP-treated WT mice. N = 4. (**B**) Immunofluorescence staining of BrdU (red) and GFAP (green) in the SVZ. Scale bar: 100 μm.

Next, we investigated if NRSF played a role in the survival of newborn cells in the SVZ of the PD mouse brain. Beginning on day 3 following the last MPTP injection, mice were injected with BrdU over a 5-day period and sacrificed 42 days after MPTP treatment. We observed that the number of BrdU (+) cells in the SVZ of saline-treated cKO mice were reduced compared to saline-treated WT mice (*p*=0.084, by unpaired T test). MPTP treatment further reduced the number of BrdU (+) cells in both genotypes, although the effect was larger in cKO mice (Figure 7A). In addition, BrdU (+) cells in the SVZ showed GFAP immunoreactivity (Figure 7B).

## DISCUSSION

The neurotoxin MPTP is commonly used to produce an irreversible and severe Parkinsonian syndrome in a variety of species ranging from non-human primates to invertebrates [28]. In the present study, PD progression and PD-related neurogenesis were investigated in whole brain *NRSF/REST* conditional knockout (cKO) and littermate WT mice. Behavioral deficits, alterations in the nigrostriatal pathway, and cellular changes in the SVZ area were assessed at early (7 days) and late (42 days) time after MPTP administration. Under baseline conditions cKO mice showed an enhanced dopaminergic system, evidenced by increased numbers of dopaminergic neurons in the SN and higher TH level and TH (+) nerve fiber density in the striatum. However, these mice displayed normal locomotor and coordinate behaviors, which may be attributable to a compensatory mechanism. MPTP caused evident short-term damage to the nigrostriatal pathway and elicited PD-associated behaviors in both genotypes. However, the loss of dopaminergic neurons, the reduction in striatal TH levels, and the behavioral deficits were more severe in cKO mice than in the littermate controls. Evidence indicates that around 2 weeks after MPTP intoxication the midbrain generally undergoes a process of recovery [21, 22, 29]. Partial recovery of dopaminergic neurons and striatal TH levels was indeed observed in all mice at 42 days post-MPTP treatment, but less so in the cKO genotype. Still, the extent of recovery was similar for WT and cKO mice, which suggests that NRSF was not critical to this process. Nevertheless, the behavioral deficits in cKO mice did not subside. Our results are consistent with the concept that there exists a certain dopamine threshold that needs to be reached to restore normal behavior in the MPTP mouse model of PD.

Neurogenesis in Parkinson patients and animal PD models is quite controversial [2, 30]. Hoglinger et al. reported a significant reduction of proliferating cell nuclear antigen (PCNA)-positive cells in the SVZ of both Parkinson patients and mice exposed to MPTP [31]. This decrease in the proliferative capacity of neural stem cells was repeatable after 6-hydroxydopamine (6-OHDA) induced dopaminergic denervation [31]. By contrast, van den Berge et al. reported that the proliferative capacity in the SVZ of both Parkinson patients and mice experiencing dopaminergic denervation induced by chronic MPTP administration was not changed or was even slightly increased [32]. This discrepancy may result from methodological differences in sample collection, animal models, and/or analysis methods between these two works. In the present study, BrdU (+) cells in the SVZ of cKO mice dramatically outnumbered those in their WT counterparts, both at baseline and 7 days after MPTP treatment. This suggests NRSF/REST acts as an inhibitor in neurogenesis under both normal conditions and during PD. On the other hand, a sharp increase in BrdU (+) cells was also seen in the SVZ of WT mice 7 days after MPTP injection, which suggested that at early PD stages neurogenesis is enhanced and is not influenced by NRSF. Thirty-four days after BrdU administration, the number of BrdU (+) cells in the SVZ of saline-treated WT and cKO mice dropped to 54% and 37.9%, respectively, of the values seen on day 7 after MPTP/saline injections. By contrast, during this time BrdU (+) cell numbers in the SVZ of MPTP-treated WT and cKO mice decreased to 35.2% and 22.3%, respectively. These results indicate that MPTP-induced PD-like microenvironment does not favor the survival of neural stem/progenitor cells, and NRSF deficiency further aggravates this situation. Hyperactivated astrocytes in the nigrostriatal pathway and impaired local trophic support may contribute to the observed death of neural progenitor cells.

One of the unmet needs in neuroscience and regenerative medicine is to understand how neurogenesis affects behavioral outcomes. Evidence has shown that increased neurogenesis in the SVZ is linked to reduced infarct volume and functional recovery in ischemic stroke [33–35]. Along these lines, strategies to enhance and sustain neurogenesis in Parkinson’s disease may be of great therapeutic potential. Chiu et al. reported that long-term treatment with L-DOPA or the dopamine agonist pramipexole restored decreased neurogenesis in the hippocampal dentate gyrus and improved non-motor behavior in a mouse PD model of bilateral 6-OHDA lesion [36]. Given the scarcity of available information, studies are warranted to address the role of intrinsic neurogenesis (especially in the SVZ) in the progression of the PD model, including early damage and late recovery of the nigrostriatal pathway, and their effects on behavior.

In summary, our study confirmed that whole-brain *NRSF/REST* conditional knockout mice were more sensitive to the neurotoxicity of MPTP than their WT counterparts, and they exhibited transiently enhanced neurogenesis early after MPTP administration. However, because of increased neuroinflammation and a lower survival rate of replicative neural progenitor cells, *NRSF/REST* depletion eventually led to decreased neurogenesis.

## MATERIALS AND METHODS

### Animal models

All experimental protocols were approved by the Institutional Animal Care and Use Committee of Fudan University, Shanghai Medical College (IACUC Animal Project Number: 20110307-055). All surgeries were performed under general anesthesia, and all efforts were made to minimize suffering.

WT mice and brain-specific conditional *NRSF* knockout (*Nestin-Cre*:*NRSF^fl/fl^*, cKO) mice (12 to 16 weeks old) were obtained from Shanghai Model Organisms Center, INC. and maintained in a temperature-controlled room under a 12 h light/ dark cycle with *ad libitum* access to food and water. All animal experiments were performed in accordance with the Guidelines of the Fudan University Animal Care and Use Committee.

### MPTP lesion

Both WT and cKO mice were divided into MPTP-treated groups and saline-treated groups. Mice in MPTP-treated groups received four intraperitoneal injections of 20 mg/kg MPTP-HCl at 2 h intervals in a single day, while control mice were injected with equal volumes of normal saline. Mice were sacrificed 1 day, 7 days or 42 days later.

### Neurogenesis studies

To assess neurogenesis, three intraperitoneal BrdU injection regimens were adopted: 1) 3 days after MPTP/saline treatment, BrdU (20 mg/kg) was injected twice daily for 5 days and then sacrificed; 2) 7 days after MPTP/saline treatment 4 BrdU (50 mg/kg) injections were applied at 2 h intervals, and mice were sacrificed 2 h later; and 3) 3 days after MPTP/saline treatment, BrdU (20 mg/kg) was injected twice daily for 5 days, and mice sacrificed 34 days later (i.e. 42 days after MPTP/saline treatment).

### Western blot analysis

Mouse tissues were lysed in T-PER reagent with a protease inhibitor cocktail (Thermo Scientific). Equal amounts of proteins were fractionated by SDS-PAGE, transferred to polyvinylidene fluoride (PVDF) membranes. Blots were probed overnight at 4°C with the following primary antibodies: rabbit anti-TH (1:4000, Sigma), mouse anti-β-actin (1:2000, Abgent), rabbit anti-GFAP (1:4000, Millipore), mouse anti-GAPDH (1:5000, Santa Cruz). Next, the membranes were incubated for 1 h with IRDye 680LT goat anti-mouse IgG (H+L) (926-68020, Li-Cor, USA) or IRDye 800CW goat anti-rabbit IgG (H+L) (926-32211, Li-Cor, USA) (1:10,000 dilutions in TBST with 0.02% SDS) and protein bands detected by an Odyssey infrared imaging system (Li-Cor, USA). Protein levels were quantified by densitometry using Quantity One 4.5.2 software (Bio-Rad, Hercules, USA).

### Immunohistochemistry and immunofluorescence

Mouse brains were fixed in 4% PFA at 4 °C overnight and cryosectioned at a thickness of 30 µm. The sections were rehydrated, blocked by 10% goat serum, and incubated with primary antibodies: mouse anti-tyrosine hydroxylase (1:1000, Sigma), mouse anti-nestin (1:500, Abcam), rabbit anti-Sox2 (1:500, Abcam), rabbit anti-Iba1 (1:500, Wako), rabbit anti-GFAP (1:1000, Millipore), and rabbit anti-BrdU (1:500, Abcam). For immunohistochemistry staining, the sections were washed and incubated with biotinylated anti-mouse or anti-rabbit secondary antibodies (1:200, Vector Laboratories, USA), and then with AB peroxidase (1:200, Vector Laboratories, USA). The peroxidase reaction was detected with 0.05% DAB (Sigma, USA) in 0.1 M Tris-HCl buffer and 0.03% H_2_O_2_. For immunofluorescence staining, the sections were then washed and labeled with fluorescent secondary antibodies (1:500, Invitrogen). Sections were mounted with ProLong Gold Antifade Reagent with DAPI (1:5000, Invitrogen), and images taken under a Leica confocal microscope (TCS SP-2, Leica, Germany).

### Densitometric analysis and cell counting

Densitometric analysis of TH-positive fibers in the striatum was performed as previously described [37]. An average of 8 sections from the striatum (bregma +1.18 mm to +0.02 mm; anteroposterior axis) were examined using Image-Pro Plus (vision 6.0, Media Cybernetics) on a computer attached to a light microscope (Leica, Germany).

To measure the density of TH-positive cells in the SNpc we performed stereological cell counting as previously described [19, 37]. Total numbers of TH-positive neurons and Nissl-stained neurons in the SNpc were counted using the optical fractionator method on a Stereo Investigator system (Micro Brightfield, USA) attached to a Leica microscope. A total of 8 sections (one every five 30 μm-thick sections collected −2.80 to −3.80 mm from bregma) were analyzed. Assays were performed in a double-blind fashion by two operators.

### Gene expression analysis using RT-PCR

Total RNA from brain tissues was extracted using the Total RNA Kit (TIANGEN, China). First-strand cDNA synthesis was performed using the FastQuant RT Kit (TIANGEN, China). The primers used for real-time PCR are shown in the Supplementary Table 1.

### Behavioral studies

#### Wire hanging test

This test is used to assess neuromuscular and locomotor development [38, 39]. Mice suspended by their forepaws from a horizontal wire (1.6 mm in diameter, 50 cm long, 30 cm high between two poles) tended to support themselves with their hind paws to avoid falling and to aid in progression along the rod. The latency to fall down and the ability to grip the wire was scored from 0 to 5 as follows: 0, animal falling immediately; 1, gripping the wire with forelimbs only; 2, gripping the wire with forepaws and trying to support itself with its hind paws; 3, gripping the wire with 3 or 4 paws; 4, gripping the wire with 4 paws and twisting its tail around the wire; 5, gripping the wire with 4 paws, twisting its tail around the wire, and moving to the pole. Maximum test time was 60 sec; latency time was recorded as 60 sec if the animal moved to the pole within this time frame.

#### Pole test

Motor function was further assessed using the pole test, which included a pole, 50 cm in height and 10 mm in diameter, with a rough surface that stood vertically in the home cage. Mice were placed near the top of the pole, heads up, and time to turn (first measurement) and climb down (second measurement) were recorded.

#### Rotarod test

Motor coordination was assessed using the rotarod test. One day before the test, mice were pre-trained on the rotarod (MED Associates, USA) for 3 times separated by 1 h intervals, using an accelerating mode (4 to 40 rpm in 5 min). On the testing day, mice ran on the rotarod at constant velocity of 16, 20, 24, 28 and 32 rpm for a maximum of 300 sec, latency to fall was recorded. Data were collected from three trials separated by 1 h intervals.

#### Open field test

Spontaneous exploratory activity and locomotion were assessed using the open field test. Test was performed using the open-field working station (MED Associates, USA). Briefly, mice were placed individually at the center of the open-field arena (40× 40× 50 cm) and the behavior of the animal was recorded using a video camera system positioned above the arena for 15 min. The total moving distance, time of ambulatory movements and resting were analyzed.

#### Statistical analysis

Statistical analyses were performed using Prism 7 (GraphPad Software Inc, USA). Comparisons were made using unpaired student’s t-test, or one-way ANOVA followed by Tukey’s multiple comparison test. All values are expressed as mean ± SEM. *P* < 0.05 was considered significant.

## Supplementary Material

Supplementary Table and Figures
