# Supplementary material for: Brain-specific NRSF deficiency aggravates dopaminergic neurodegeneration and impairs neurogenesis in the MPTP mouse model of Parkinson’s disease
Source: Aging (Albany NY). 2019 May 30;11(10):3280–97. doi: 10.18632/aging.101979 (PMC6555471; doi:10.18632/aging.101979)
Supplement: Supplementary Table and Figures [file aging-11-101979-s001.pdf]

SUPPLEMENTARY MATERIAL

Supplementary Table 1. The primers used for real-time PCR.

Primer	Forward（5'-3'）	Reverse（5'-3'）
β-actin	CAGGATGCAGAAGGAGATTAC	AACGCAGCTCAGTAACAGTC
Bax	GGATTGTGGCCTTCTTTGAGTTCGG	CATATTTGTTTGGGGCAGGTTTGTC
Bid	GTGCAAGCTTACTGGGAGG	TATTTGGGCGAGATGTCTGG
Fas	AAGTCCCAGAAATCGCCTATG	GGTATGGTTTCACGACTGGAG
IL-1α	GCAACTGTTCCTGAACTC	CTCGGAGCCTGTAGTGCA
IL-1β	ACGGACCCCAAAAGATGAAG	TTCTCCACAGCCACAATGAG
IL-6	GATGCTACCAAACTGGATATAATCAG	CTCTGAAGGACTCTGGCTTTG
Mapk11	TGTCTCGCCCTTTCCAATC	AGGTACACTTCGCTGAAATCC
TNF-α	CACGCTCTTCTGTCTACTGAACTTC	GCAGCCTTGTCCCTTGAAGAGAACC
iNOS	CCCTTCCGAAGTTTCTGGCAGCAGC	GGCTGTCAGAGCCTCGTGGCTTTGG

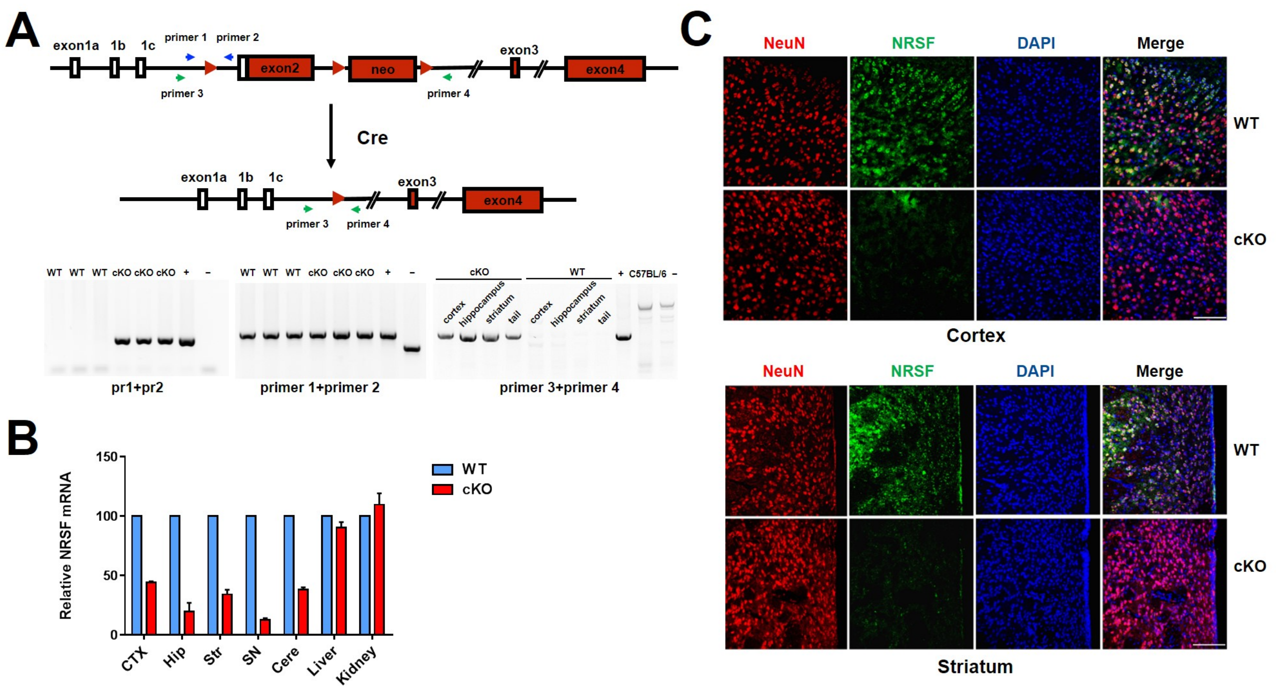

Supplementary Figure 1. Genetic ablation of NRSF/REST in the brain of mice. (**A**) Gene targeting strategy and the positive targeting event. Primers pr1 and pr2, primer 1 and primer 2, primer 3 and primer 4 were used to identify *Nestin-Cre* transgene, mouse genomic DNA before and after recombination, respectively. (**B**) Quantitative RT-PCR analysis of relative *NRSF* mRNA level in cKO and WT mice. Data are mean ±SEM. CTX: cortex; Hip: hippocampus; Str: striatum; SN: substantia nigra; Cere: cerebellum. (**C**) Brain sections containing the cortex or striatum were stained with antibodies against NeuN (red) and NRSF (green). Scale bars: 100 μm.

The primer sequences are:

pr1: 5′—cttggctttgtactttctgtgactg—3′

pr2: 5′—cctccatcccagacaaatacattac—3′

primer 1: 5′—tgctcattagaggccccagaatcg—3′

primer 2: 5′—tggcagtactcgcatgtaatccta—3′

primer 3: 5′—ccgtagccaccccttaagagtcatc—3′

primer 4: 5′—ggccagtctgagctcctagagatcc—3′

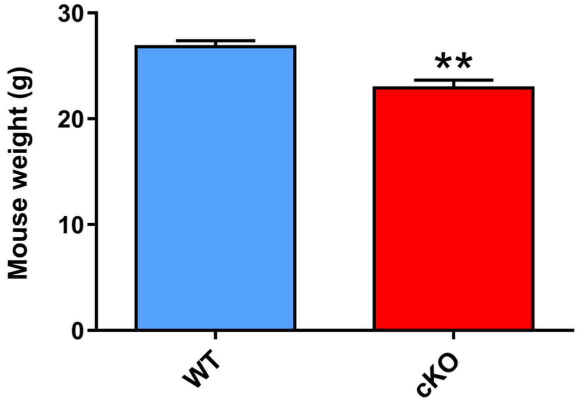

Supplementary Figure 2. Weight of WT and cKO mice at the age of 12-16 weeks. Data are means ±SEM. $^{**}p<0.01$. N = 48 for WT mice and N = 32 for cKO mice.

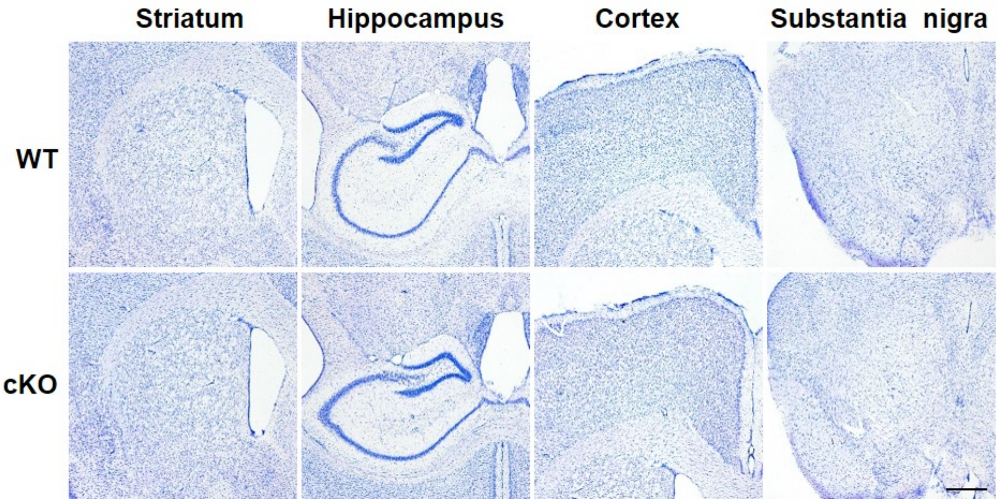

Supplementary Figure 3. Creysl violet staining of brain sections of WT and cKO mice. Scale bar: 100 µm. The sections (30 µm thick) were fixed for at least 24 h by immersion in 4% paraformaldehyde in 0.1 M phosphate buffer saline (PBS, pH 7.2) and washed with distilled water. Then the sections were covered with filtered cresyl violet stain for 15 min, dehydrated with ascending grades of alcohol, cleared with xylene, observed and photographed under Olympus microscope (BX53, Japan).

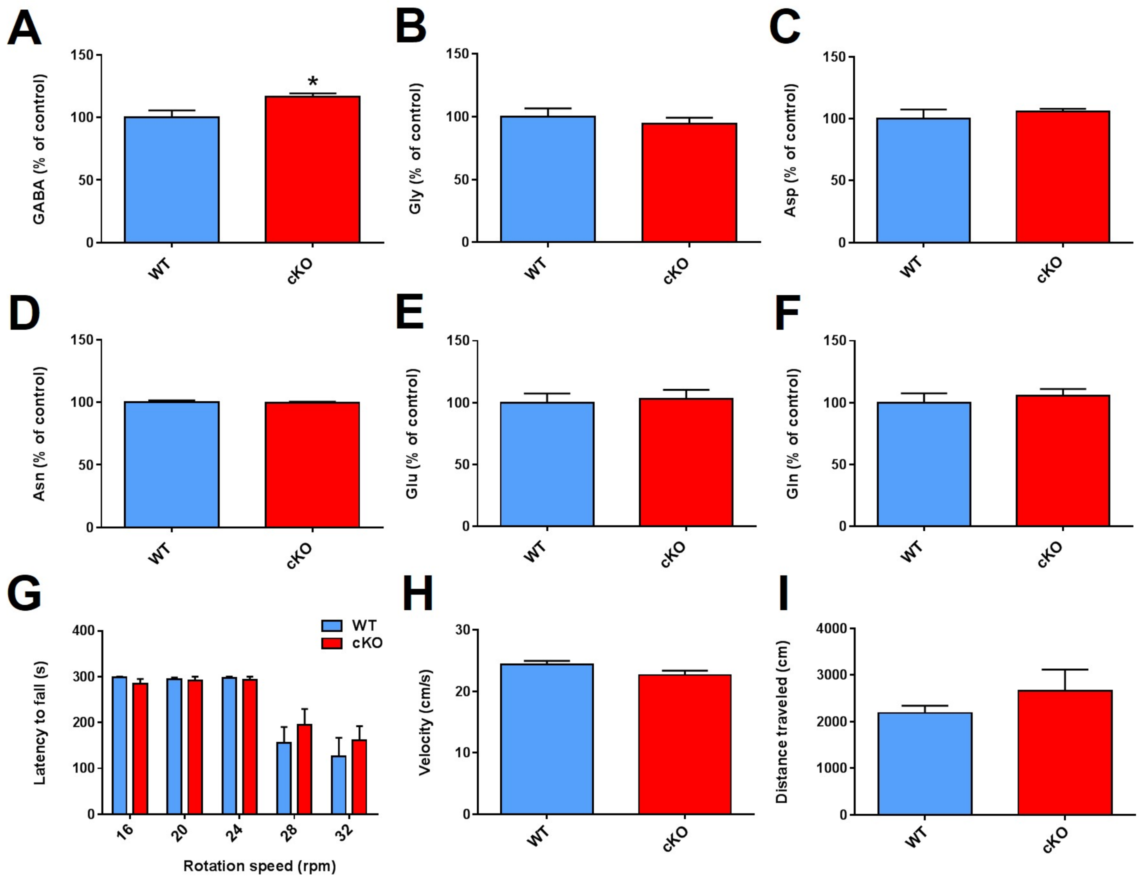

Supplementary Figure 4. The striatal concentrations of amino acid neurotransmitters analyzed by HPLC assay (**A**–**F**) and the effects of the brain deficiency of NRSF/REST on motor behaviors (**G**–**J**). (**A**) GABA; (**B**) Glycine; (**C**) Aspartate; (**D**) Asparagine; (**E**) Glutamate; (**F**) Glutamine. N = 5. (**G**) Length of time mice stayed on the rod at different speeds in the rotarod test. N = 9. (**H**, **I**) Open field test results. (**H**) Velocity. (**I**) Distance traveled during a period of 15 min. N = 14. Data are means ± SEM. Differences were analyzed by Student's t-test. * $p < 0.05$.

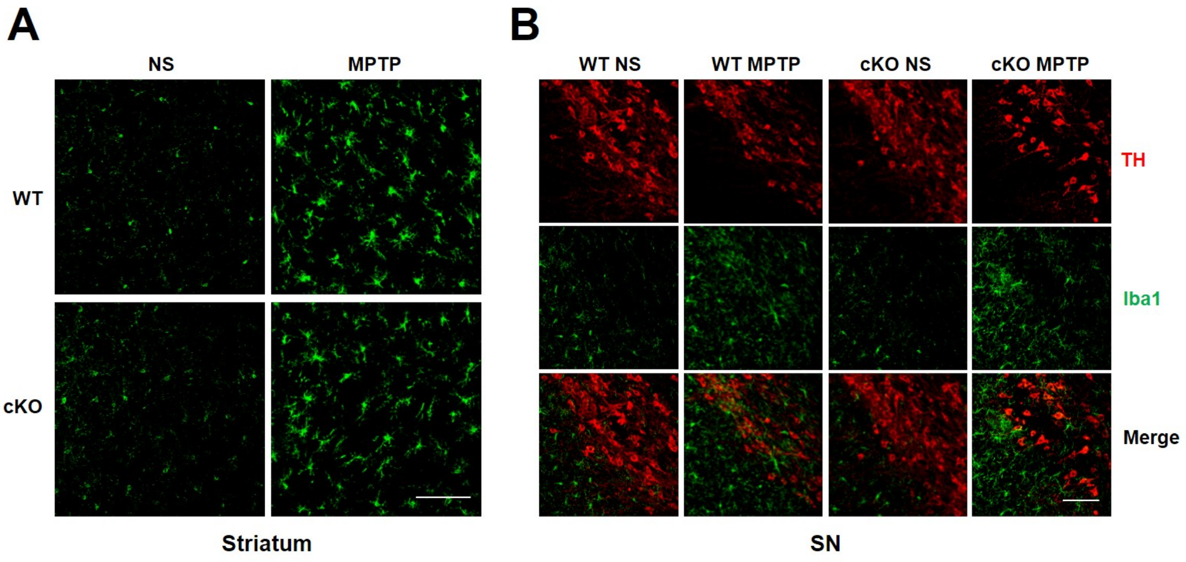

Supplementary Figure 5. Deletion of NRSF in the brain did not affect MPTP-induced microglial activation in the nigrostriatal pathway at 1 day post-injection. (**A**) Immunofluorescence staining of Iba1 (green) in the striatum of WT and cKO mice. Scale bar: 200 µm. (**B**) Immunofluorescence staining of TH (red) and Iba1 (green) in the substantia nigra of WT and cKO mice. Scale bar: 100 µm.

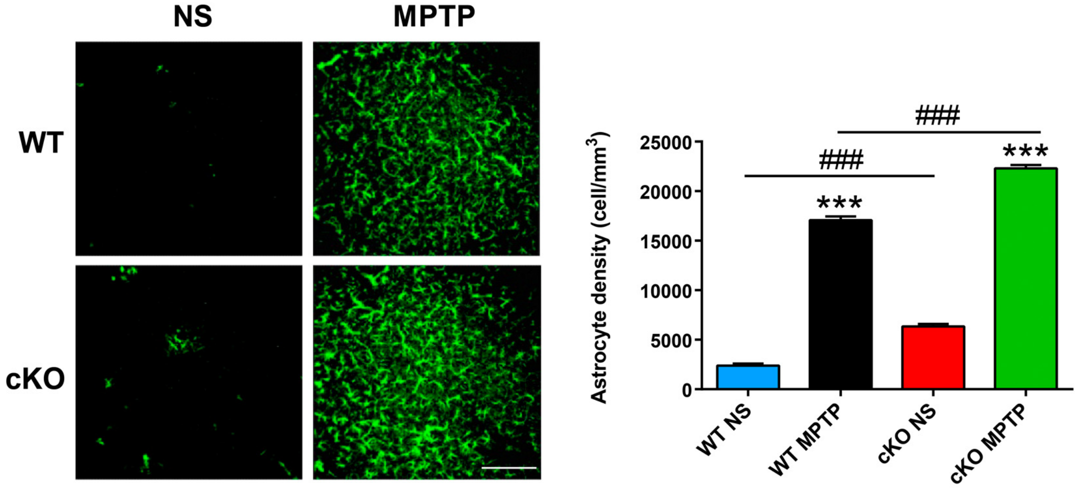

Supplementary Figure 6. Astrocyte activation in the striatum of WT and cKO mice at 7 days after MPTP/saline treatment. Immunofluorescence staining of GFAP (green) in the striatum of WT and cKO mice. Scale bar: 100 µm. Quantification of GFAP-positive cells is showed in the right panel. Data are means ±SEM. Differences were analyzed by one-way ANOVA. ***p<0.001, vs saline-treated control; ### p<0.001, vs MPTP-treated WT mice. N = 5-7.

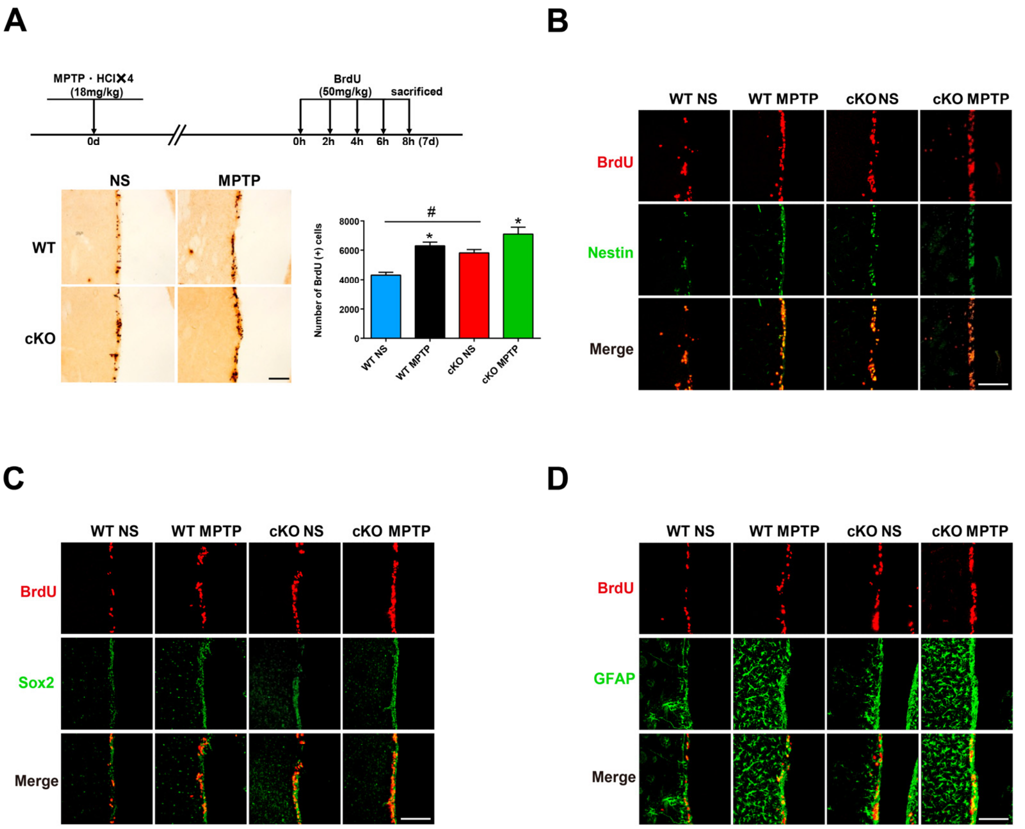

Supplementary Figure 7. Neurogenesis in the SVZ of WT and cKO mice at 7 days after MPTP/saline treatment. Four times of BrdU injection were conducted with 2 hour intervals at day 7. (**A**) Immunohistochemistry staining of BrdU in the SVZ. Scale bar: 200 μm. Quantification of BrdU-positive cells is showed in the right panel. Data are means ±SEM. Differences were analyzed by one-way ANOVA. *$p<0.05$, vs saline-treated control; # $p<0.05$, vs MPTP-treated WT mice. N = 3-4. (**B**–**D**) Immunofluorescence staining of BrdU (red) and Nestin (green), Sox2 (green) or GFAP (green) in the SVZ. Scale bar: 100 μm.
